# Shiny-Seq: advanced guided transcriptome analysis

**DOI:** 10.1186/s13104-019-4471-1

**Published:** 2019-07-18

**Authors:** Zenitha Sundararajan, Rainer Knoll, Peter Hombach, Matthias Becker, Joachim L. Schultze, Thomas Ulas

**Affiliations:** 1 Genomics and Immunoregulation, LIMES Institute, University of Bonn, Carl-Troll-Str. 31, 53113 Bonn, Germany; 2 Platform for Single Cell Genomics and Epigenomics (PRECISE) at the German Center for Neurodegenerative Diseases and the University of Bonn, Venusberg-Campus 1, Gebäude 99, 53127 Bonn, Germany

**Keywords:** RNA-Seq, Bioinformatics, Analysis, Shiny, DeSeq2, Functional prediction, Limma, Co-expression network analysis, Pipeline, Automated report

## Abstract

**Objective:**

A comprehensive analysis of RNA-Seq data uses a wide range of different tools and algorithms, which are normally limited to R users only. While several tools and advanced analysis pipelines are available, some require programming skills and others lack the support for many important features that enable a more comprehensive data analysis. There is thus, a need for a guided and easy to use comprehensive RNA-Seq data platform, which integrates the state of the art analysis workflow.

**Results:**

We present the tool Shiny-Seq, which provides a guided and easy to use comprehensive RNA-Seq data analysis pipeline. It has many features such as batch effect estimation and removal, quality check with several visualization options, enrichment analysis with multiple biological databases, identification of patterns using advanced methods such as weighted gene co-expression network analysis, summarizing analysis as power point presentation and all results as tables via a one-click feature. The source code is published on GitHub (https://github.com/schultzelab/Shiny-Seq) and licensed under GPLv3. Shiny-Seq is written in R using the Shiny framework. In addition, the application is hosted on a public website hosted by the shinyapps.io server (https://schultzelab.shinyapps.io/Shiny-Seq/) and as a Docker image https://hub.docker.com/r/makaho/shiny-seq.

## Introduction

The scientific community is continuously trying to improve their understanding of genetic mechanisms in biological systems in a global way. Particularly transcriptome analysis has become an everyday research tool to study the regulation and the function of complete genomes [1]. Here, Next Generation Sequencing (NGS) has become one of the preferred methods. Constantly dropping sequencing costs and more than 25,000 (ArrayExpress, NCBI GEO) publically available transcriptome datasets help us to better understand the complex relationship between genotype and phenotype. With growing accessibility, still, only the minority of investigators in the life and medical sciences has the means to analyze and leverage this enormous treasure of data. Understanding RNA-Seq data requires several successive steps in order to analyze, visualize and interpret it. The key steps are (i) import of data, (ii) normalization, (iii) analysis using statistical techniques such as hypothesis testing, (iv) functional enrichment analysis using various biological databases, and (v) identification of biological patterns using advanced methods such co-expression network analysis. Integrated, simply accessible, easily expandable and inexpensive tools are still missing. Shiny-Seq is providing such an analysis environment for the broader community in the life and medical sciences.

## Main text

### Shiny-Seq section

In the following, we provide details regarding features implemented in the various steps of Shiny-Seq. The main text consists of three different sections: data pre-processing (1), exploratory data analysis (2), and downstream analysis (3) and its respective subsections.

#### Data pre-processing

##### Input

Our Shiny-Seq pipeline provides two different starting points for the analysis. First, the count table, which is the universal file format produced by most of the alignment and quantification tools. Second, the transcript-level abundance estimates provided by ultrafast pseudoalignment tools like *kallisto* [2]. For this purpose, the user has to provide the location of the directory containing the files generated by *kallisto*. Another essential input is the annotation file, a matrix that stores for each sample different categorical variables e.g. treatment, genotype, sex or day of the experiment.

##### Normalization

The package *DESeq2* [3] normalizes the dataset by computing a size factor for each sample. The size factor is calculated by taking the median ratio of each sample over a reference or pseudo sample. Shiny-Seq uses the default parameter recommended by the Bioconductor DESeq2 workflow for RNA-Seq [4] data but also allows to control for log_2_ fold change shrinkage and multiple testing, custom p-value and fold change cut-offs.

##### Batch effect analysis

Batch effects can be induced by either known variables such as technical heterogeneity and time of experiment or by unknown variables [5]. In Shiny-Seq, the function *removeBatcheffect* from *LIMMA* [6] is used to account for the batch effect from known sources. For unknown variables, Shiny-Seq uses *SVA* [5] to construct surrogate variables to account for technical variability. The influence of potential variables known to cause the batch effect can then be examined by PCA. The detected batch effects are modeled within the *DESeq2* study design and the batch corrected data is used for all respective visualizations.

Additionally, Shiny-Seq can estimate the influence of the batch effect based on an ANOVA model and visualize it via a source of variation plot showing the effects sizes of the modeled factors.

#### Exploratory data analysis

##### Differential gene expression analysis

Shiny-Seq supports *DeSeq2*’s differential gene expression testing (DGEA) based on a negative binomial distribution model. *DeSeq2* uses variance-mean estimation for RNA-Seq data and the Wald test. The Wald test assumes that the Z-statistic takes a standard normal distribution with zero mean and unit variance. Additionally, Shiny-Seq supports p-value evaluation and correction, where a histogram is generated, which helps to decide whether the statistical hypothesis assumption is violated. If necessary, the correction can be performed using *fdrtool* [7].

##### Co-expression network analysis

In contrast to conventional DGEA, Shiny-Seq also provides a co-expression network analysis (CENA) function using WGCNA [8]. This method allows identifying modules based on correlation followed by network analysis. It takes the pre-processed data and the annotation file as inputs but can also take results from the DEGA as a starting point. Note that batch corrected data is used as input for the CENA if a batch correction was selected beforehand. The output is the typical module-condition relationship heat map and a table including module name, number of genes and identified hub genes in each module. Furthermore, the identified modules are integrated into Shiny-Seq in a way that the user can perform the major parts of the downstream analysis e.g. functional enrichment analysis, heat maps, and Venn diagrams based on these results.

#### Downstream analysis

##### Functional prediction

After DGEA and CENA a functional prediction based on gene set enrichment analysis (GSEA) can be performed. Shiny-Seq uses biological databases such as KEGG [9], GO [10] and Broad’s molecular signatures database (MSigDB) [11] in *clusterprofiler*’s [12] GSEA to take advantage of already publicly available knowledge, which assists during the interpretation process. Shiny-Seq uses FDR correction by the Benjamini and Hochberg method, which reduces the proportion of false positive results significantly.

##### Transcription factor binding side overrepresentation analysis

Our application also performs a transcription factor binding site overrepresentation analysis in the promoter regions for all groups of genes being identified by DGEA and CENA. This analysis generates a table with information like enrichment p-value about potential transcription factor binding sites discovered by searching promoters databases TRANSFAC [13] and Jaspar [14] in human or mouse, respectively. All predicted transcription factors are displayed in a new table and are additionally marked in the table of differentially expressed genes. This analysis provides valuable information about potential upstream regulators responsible for the observed genotype. Shiny-Seq uses *pcaGopromoter* [15] to predict transcription factors.

##### Visualization

**Fig. 1:**
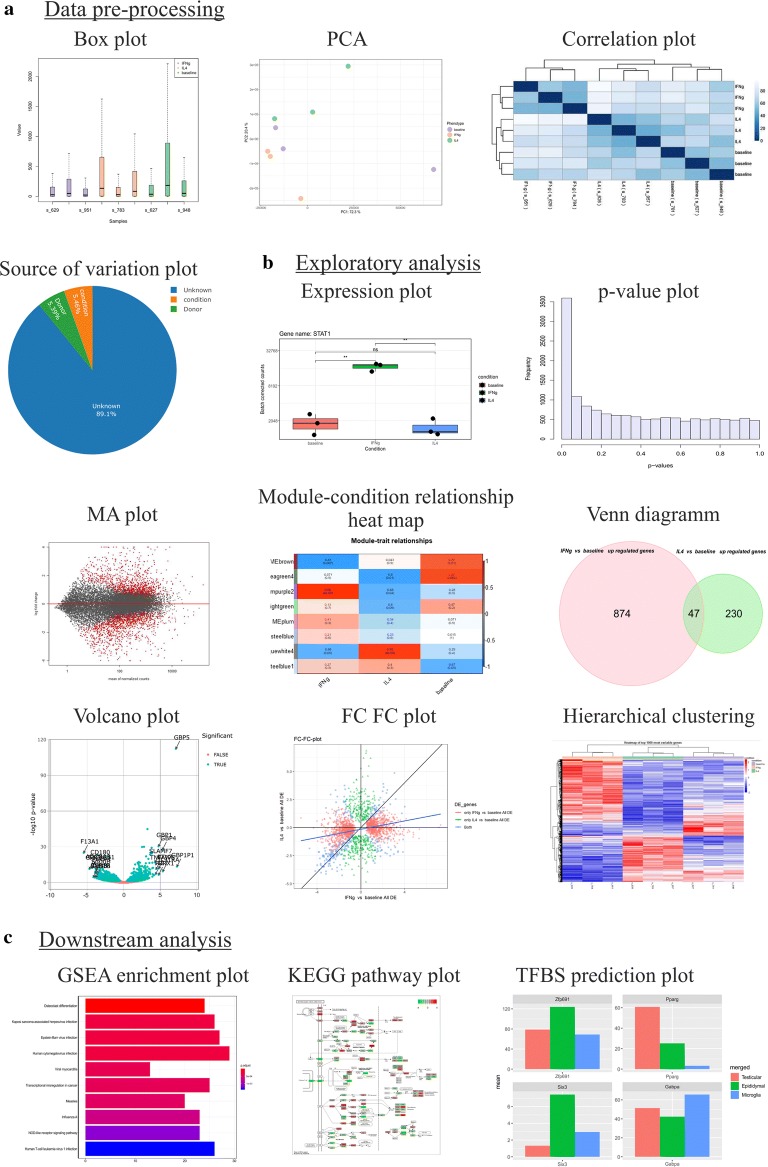
Data pre-processing (**a**) box plots of samples (before and after normalization), PCA (2D and 3D) of samples (before, after normalization and after batch correction; interactive), sample correlation plot (before and after batch correction), source of variation plot (before and after batch correction; interactive); Exploratory analysis (**b**): box plot of single gene expression including statistics, p-value evaluation histogram, MA plot, module-condition relationship heat map (CENA), Venn diagram (interactive), volcano plot (interactive), fold change fold change plot (interactive), heatmap of 1000 most variable genes, own gene list, DEGA and CENA results; Downstream analysis (**c**): dot plots of GSEA results (interactive), visualization of KEGG pathways (DEGA genes or all present genes), TFBS plot

Shiny-Seq provides a multitude of visualizations in the respective analysis steps (Fig. 1). This includes plots such as heat maps and volcano plots, which are commonly used during the analysis of RNA-Seq data. A heat map, for example, visualizes relationships between samples and genes. Shiny-Seq uses heat maps for the visualization of differentially expressed genes, 1000 genes having the highest variance within the data and all present and differential expressed transcription factors. Volcano plots help to visualize differentially expressed genes obtained from DGEA. While heat maps and volcano plots are used to visualize e.g. hypothesis test results of a single comparison, they do not have the capability to compare results obtained from multiple comparisons. To tackle this Shiny-Seq is providing Venn diagrams and a fold change fold change plots, where the names of genes of interest can be identified by selecting them interactively in the respective plot. Static plots e.g. heat maps can be download as vector graphic for further usage. If meaningful, some of the plots can be further customized within Shiny-Seq.

#### Report generation

Another unique feature is the compilation of all outputs generated during each step of the analysis and summarizing these results in a PowerPoint presentation, as well as respective tables, which can be downloaded and shared with colleagues and collaborators. It includes QC plots e.g. box plots and PCA plots before and after normalization, top-10 up-regulated and down-regulated genes, and enrichment analysis results. The R package *ReporteRs* [16] is used to generate a presentation.

### Discussion

Global transcriptome analysis has become a standard approach in research but also in clinical settings. At the same time, experts who can analyze this kind of data are still the limiting factor. Shiny-Seq provides a framework for analyzing such data in a transparent and reproducible manner for NGS service providers and NGS competence centers, but also for end users with limited scripting experience. It offers a huge functionality combined with a guided and intuitive workflow and a comprehensive and time-saving summary functionality. Providing Shiny-Seq as a fully functional Docker image, there is no need to install R. The code, all packages, and their dependencies are installed within the Docker image and this is available on Docker Hub. By using Shiny as graphical interface, the user does not need any computer or programming skills.

## Limitations

While the development is complete from the end-user perspective, the internally used R code is still cluttered. Moreover, incorporation of new features and additional customization of the visualizations would further improve Shiny-Seq. The application currently supports only enrichment analysis of gene ontologies, pathways, and molecular signatures. The plan is to extend the support to disease ontologies as well. In the future, Shiny-Seq will get the capability to support also count tables from transcript quantification files generated by other tools such as Star [17], HTSeq-counts [18], and Sailfish [19]. The export of a DESeq2 RData object would provide more flexibility for users with programming experience.

## Data Availability

All data in this study were included in this article.

## Abbreviations

DGEA: differentially genes expression analysis
CENA: co-expression network analysis
GO: gene ontology
GSEA: gene set enrichment analysis
KEGG: Kyoto Encyclopedia of Genes and Genomes
MSigDB: molecular signatures database
NGS: Next Generation Sequencing
PCA: principal component analysis
QC: quality control
WGCNA: weighted gene co-expression network analysis
